# Risk of Second Cancers in Merkel Cell Carcinoma: A Meta-Analysis of Population Based Cohort Studies

**DOI:** 10.1155/2014/184245

**Published:** 2014-12-10

**Authors:** Anshul Saxena, Muni Rubens, Venkataraghavan Ramamoorthy, Hafiz Khan

**Affiliations:** ^1^Department of Health Promotion and Disease Prevention, Robert Stempel College of Public Health and Social Work, Florida International University, Biscayne Bay Campus, ACI-260, 3000 NE 151st Street, Miami, FL 33181, USA; ^2^Department of Dietetics and Nutrition, Florida International University, Miami, FL 33199, USA; ^3^Department of Biostatistics, Florida International University, Miami, FL 33199, USA

## Abstract

The risk of second cancers in Merkel cell carcinoma (MCC) remains uncertain since risk estimates vary worldwide. The global MCC population is growing and there is a demand for better knowledge of prognosis of this disease. The Cochrane Database of Systematic Reviews, MEDLINE, and EMBASE search engines were searched for the relevant literature between January 1999 and September 2014 by use of explicit search criteria. The main outcome was second malignancies associated with MCC patients measured by standardized incidence ratios (SIRs) or other estimates of risks. Five papers fulfilled the inclusion criteria and reported SIRs of second cancer in MCC which varied from 1.07 to 2.80. Performing meta-analysis using random effects model revealed that there was an increased risk for second malignancies due to MCC (SIR, 1.52; 95% CI, 1.10–2.11). There was a significant increase in risk for malignant melanoma (SIR, 3.09; 95% CI, 2.02–4.73) as compared to all common second malignancies among the studies. Updated knowledge about risk of second malignancies in MCC will help in better assessment of the disease prognosis and will help in optimizing the medical and surgical treatment, radiotherapy, follow-up, and surveillance procedures.

## 1. Introduction

Merkel cell carcinoma (MCC), a rare and aggressive neuroendocrine tumor, was first reported in 1972 as a variant of “trabecular carcinoma of the skin” [1]. Studies based on Survival Epidemiology and End Result Program (SEER) have shown that estimated age-adjusted incidence rate for MCC was as low as 0.18 to 0.41 per 100,000 population by the year of 2006 [2]. Subsequent studies have estimated a fourfold increase in incidence over the last two decades [3, 4]. Prognosis of this cancer is very poor because this cancer grows rapidly and has high risk for early metastasis [5, 6]. MCC is essentially the tumor of Merkel cells, which are cells derived from the proliferative keratinocyte layer of skin [7–9]. Exposure to ultraviolet (UV) radiation, reactive oxygen species, and arsenic as well as immunosuppression are some of the known risk factors for this malignancy [10–12]. Some studies also suspect a rare polyomavirus as the probable causative agent [13]. This virus was demonstrated in 80% of all MCC tumors and was successively named Merkel cell polyomavirus (MCPyV) [14].

Currently, biopsy is the main procedure for diagnosis of MCC. Immunohistochemical staining and electron microscopy have greatly advanced the diagnostic accuracy [15]. MCC stains like neuron-specific enolase, synaptophysin, chromogranin, cytokeratin 20, and CAM 5.2 have also aided in better diagnosis of the condition [16]. Due to rarity of the tumor, there are very few evidence based treatment protocols, and surgical excision and radiotherapy are considered standard treatment options [17]. Though MCC is a locally aggressive malignant tumor, there are reports of distant metastasis and recurrences following treatment modalities like surgery and radiotherapy [18–20].

Studies have shown associations between diagnosis of MCC and cancers of distant organs like brain, salivary gland, and biliary tract [21, 22]. Other malignancies like multiple myeloma, chronic lymphocytic lymphoma (CLL), basal cell carcinoma of the skin, and non-Hodgkin's lymphoma (NHL) have also shown positive correlation with MCC [4, 23]. In spite of these associations, there are very few studies that have calculated the risk for second malignancies among MCC patients. A better knowledge of disease prognosis with regard to second malignancies is important for optimizing the medical and surgical management, radiotherapy, follow-up, and surveillance procedures for this condition. Management of second cancers associated with MCC can go a long way with identification of pooled risks for several common second cancers associated with the disease. The specific aim of this meta-analysis was to gather all available studies and provide quantitative estimates of the risks for many second malignancies due to MCC. The objectives of the study were (1) to perform a meta-analysis of overall risks for second malignancies after one year of established diagnosis of MCC; (2) to perform a metaregression analysis to understand the influence of latitude, mean follow-up time for second malignancies in MCC, and publication year; and (3) to evaluate the risk for many isolated second malignancies due to MCC.

## 2. Methods

### 2.1. Literature Searches

In order to identify studies for second malignancies due to MCC, we searched MEDLINE, the Cochrane Database of Systematic Reviews, and EMBASE for studies with well-defined population cohorts. We used MeSH terms and combination of text words such as “Merkel cell,” “Merkel cell carcinoma,” “MCC,” “second malignancies,” “neoplasia,” and “neuroendocrine tumors.” In the second step, these keywords were combined using the Boolean operator “and” and “or” with the terms “standardized incidence ratio,” “standardized mortality ratio,” and “risk ratio.” In addition, we searched the references lists of relevant studies to find more published articles. We also looked for recently published abstracts using PubMed search. We limited our search results to include only human studies.

### 2.2. Inclusion and Exclusion Criteria

We included studies that met each of the following criteria: (i) published in English language between January 1999 and September 2014; (ii) patients with all stages of MCC; (iii) second malignancies in MCC cases; (iv) studies which reported risk ratio, standardized incidence ratios (SIRs), or data allowing similar outcomes to be derived; (v) published as original papers (no reviews, comments, letters, or editorials); (vi) studies which reported the total number of patients with MCC and second malignancies occurring in the cohort during follow-up; (vii) studies which reported expected cancer incidence rates in a matched background population and/or rates of observed-to-expected cancers with at least 90% confidence interval (CI). Studies on only cancer mortality and referral-center studies reviews were excluded. In case of duplicate publications, the paper providing the longest follow-up of patients was used.

### 2.3. Data Extraction and Analysis

Two authors (Muni Rubens and Venkataraghavan Ramamoorthy) reviewed all potentially relevant manuscripts to determine the studies which met inclusion criteria. Data extracted from independent studies were cross-checked by three reviewers (Anshul Saxena, Muni Rubens, and Hafiz Khan); disagreements were resolved by consensus among all the authors. Extracted data included paper characteristics (first author's last name, publication year, country in which the study was carried out, and data source), study design, number of MCC patients, mean or median age of patients, duration of follow-up, and number of cases observed with second malignancies. We used STATA software program (StataCorp., College Station, TX) to calculate pooled SIRs [29]. We used *I* ^2^ statistic (0%–100%) to assess study heterogeneity for determining whether a random or fixed effects model would be appropriate for determining the pooled SIRs for different sets of second malignancies [30]. Then we did metaregression analyses to understand the influence of year of publication, mean follow-up years, and latitude of study location. Finally, we performed the meta-analysis for estimation of pooled risk for all second malignancies, as well as specific malignancies after one year of diagnosis because most studies reported SIRs for second malignancies at one year.

## 3. Results

**Figure 1:**
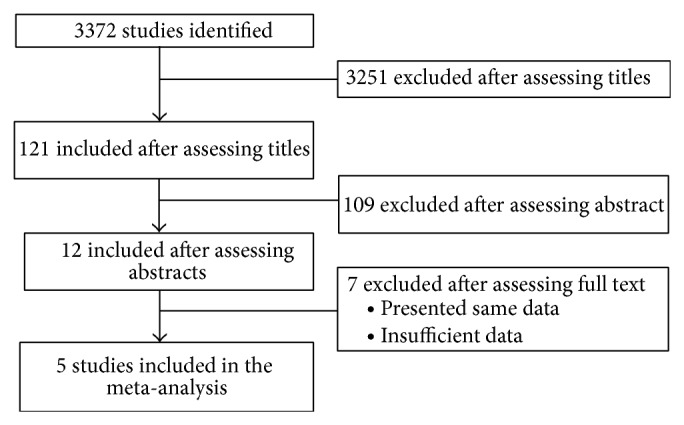
Flowchart for study selection.

There were five studies with a total of 3,098 cases, which fulfilled the inclusion criteria for meta-analysis (Figure 1 and Table 1) [24–28]. These five studies included MCC cases from North America, Scandinavia, Middle East, Australia, and Southern Europe. All the five studies reported nearly equal number of observed and expected second malignancies due to MCC. SIRs for all the five studies ranged between 1.07 (95% CI, 0.85–1.33) and 2.80 (95% CI, 1.38–4.22). The SIRs for two studies were almost similar, whereas for one study they were relatively high. For the remaining two studies the SIRs were close to one another (Table 1). The highest mean follow-up time was 4.1 years among these five studies. Only one study showed evidence of treatment for MCC using modalities like surgery, radiotherapy, and chemotherapy [25].

**Figure 2:**
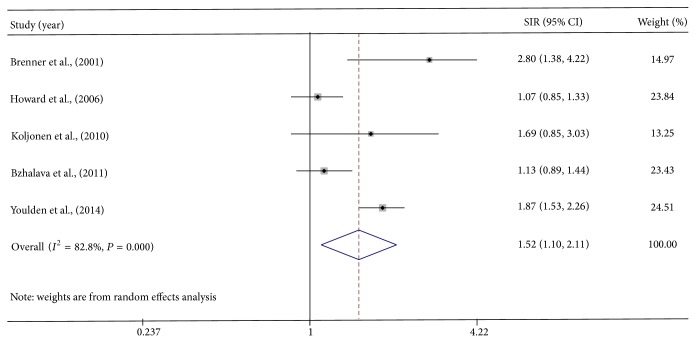
MCC and the risk of second cancer. Squares indicate the odds ratios for the individual studies; horizontal lines indicate the 95% confidence interval. The size of the data marker corresponds to the relative weight assigned in the pooled analysis using the random effects model. Diamond indicates the pooled odds ratios with 95% confidence interval.

Data were pooled using random effects model because of the significant study heterogeneity (*I* ^2^ = 83%). Random effects model for estimation of risks revealed that there was increased risk for second malignancies due to MCC (SIR, 1.52; 95% CI, 1.10–2.11) (Figure 2). Excluding the study reporting the highest SIR for second malignancies from the random effects model did change the result (SIR, 1.37; 95% CI, 1.0–1.89). On the contrary, excluding the study reporting the lowest SIR significantly increased the risk for second malignancies in MCC (SIR, 1.70; 95% CI, 1.17–2.47). Including the three studies with similar SIRs also showed increased risk for second malignancies due to MCC (SIR, 1.51; 95% CI, 1.02–2.23).

**Figure 3:**
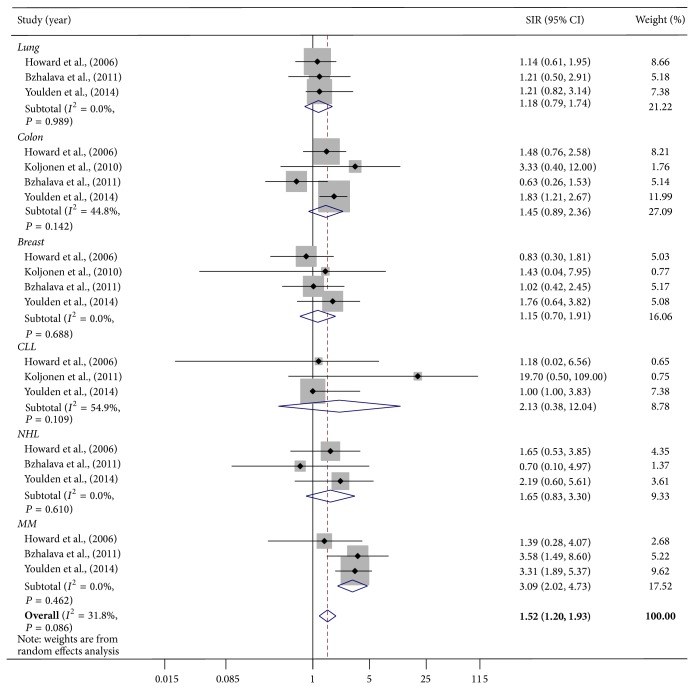
Risk of second cancer after one year of MCC diagnosis. Squares indicate the odds ratios for the individual studies; horizontal lines indicate the 95% confidence interval. The size of the data marker corresponds to the relative weight assigned in the pooled analysis using the random effects model. Diamond indicates the pooled odds ratios with 95% confidence interval. NHL: non-Hodgkin's lymphoma; CLL: chronic lymphocytic lymphoma; MM: malignant melanoma.

Most studies reported the SIRs for individual malignancies. We also performed separate risk estimates for individual second malignancies that were common in at least three of the studies (Table 2). We pooled the SIRs for breast and colon cancers from four studies and the SIRs for lung cancer, non-Hodgkin's lymphoma (NHL), chronic lymphocytic lymphoma (CLL), and malignant melanoma from three studies. Pooled SIR for malignant melanoma was significantly higher than the rest of the common malignancies (SIR, 3.09; 95% CI, 2.02–4.73). Colon cancer (SIR, 1.45; 95% CI, 0.89–2.36), CLL (SIR, 2.13; 95% CI, 0.38–12.04), NHL (SIR, 1.65; 95% CI, 0.83–3.30), lung cancer (SIR, 1.18; 95% CI, 0.79–1.74), and breast cancer (SIR, 1.15; 95% CI, 0.69–1.91) showed increased risks when compared to all second malignancies, though none of them were significant (Figure 3).

We also performed analysis using random effects model to determine the pooled risk for the combination of six second malignancies that were common in at least three of the studies. We obtained a total of 20 pooled SIRs. Random effects model estimate revealed that there was a significant increase in risk for these six second malignancies when compared to the grand total of all malignancies (SIR, 1.52; 95% CI, 1.20–1.93). The risk did not increase from 52% for these common six malignancies when compared to all second malignancies.

Metaregression analyses to understand the effects of mean follow-up years, geographic location (latitude), and publication year on the risk for all second malignancies due to MCC showed that, after allowing for additive residual heterogeneity, there was no significant association between geographic location, mean years of follow-up, and publication year and second malignancies. However, the SIR and absolute latitude showed negative relationship; that is, the higher the absolute latitude, the lower the SIR, and hence the lower the risk of second cancers (*P* > 0.05). The year of publication (*P* > 0.05) and mean follow-up years (*P* > 0.50) showed positive relationship; that is, the higher the mean follow-up period or the year of publication, the higher the chances of reporting a second cancer.

## 4. Discussion

This could be the first meta-analysis of second malignancies in MCC. We have restricted our search to include population based studies because such studies are the best fit for overall analysis of associated risks for many diseases. Many of the individual studies assert that being diagnosed with MCC poses an increased risk of being diagnosed with second primary cancer. Our results are consistent with previous reports that people with MCC are at a significantly higher risk for developing second malignancies when compared to the general population, and such risk would be present 1 to 5 years after having MCC as primary cancer. Our study asserts that patients who are diagnosed with MCC are 52% more likely to develop second cancers after one-year period as compared to the general population. The risk of developing malignant melanoma was two times more likely in patients with MCC as compared to other common malignancies. Thus we derived that, after diagnosis of MCC, there is a need to emphasize on the screening and prevention of second cancers along with the treatment of MCC.

Studies that were conducted before 10 years showed increased SIRs for second malignancies due to MCC, whereas studies that are recently conducted show decreased risk for second malignancies. This trend could be ascribed to better diagnostic and therapeutic procedures which in turn could have decreased the risk for second malignancies [31]. Assessment for the association between treatment exposure and risk for second malignancies was not performed as only one study reported exposure to treatment [25].

Our study faces some limitations such as the methodology of studies which was not assessed considering the shortcomings of quality scoring in observational studies, failure to include studies in languages other than English, large heterogeneity among individual studies, and extremely large duration of observed period in individual studies (1979–2010) [32–34]. The long number of years of study duration was a limitation because many advances have happened in both diagnostic procedures as well as therapeutic regimens for many different types of cancers during these years. Another impediment was that many specific cancers were grouped differently in individual papers thereby making comparability difficult. In addition, other factors that acted against our meta-analysis include small number of studies, restricted details in the selected studies, and lack of comparability factors due to rarity of the disease itself. There were wide variations in the risk estimates in individual studies as reflected in the differences between SIRs for total second malignancies due to MCC. This could be due to selection biases and other limitations associated with individual studies themselves.

The strengths of our study include use of standardized tools to retrieve studies as well as standard meta-analysis procedures to pool data. Our studies cover wide geographical areas from Asia to Middle East, Australia, North America, and Scandinavia. Incidentally, we observed that latitude played a role in increasing the risk for second malignancies due to MCC. However, this finding was not significant. There should be further studies on the relationship between exposure to UV radiations and risk for having second malignancy due to MCC.

Several explanations can be given for the increase for second malignancies in MCC that we observed. MCC is an extremely aggressive cancer with 5-year survival rates ranging from 68%, 52%, and 17% for local disease, regional disease, and distant metastasis, respectively [2]. Many of the second malignancies associated with MCC are slow growing tumors and therefore are masked by the effects of MCC itself.

## 5. Conclusion

Our results demonstrate that there is overall increased risk for second malignancies one year after the diagnosis of MCC. Malignant melanoma alone had two times more risk when compared to other second cancers after one year of diagnosis of MCC. The complexity of MCC involves several factors like genetic susceptibility, environmental factors, and biological factors, thereby warranting further studies with greater number of variables.

## Figures and Tables

**Table 1:** Characteristics of studies included in the meta-analysis.

Study, year	Country	Data source, study period	Number of cases of MCC	Age at diagnosis (*n* or median)	Mean follow-up	Number of second malignancies	All malignancies SIR (95% CI)	Number solid of cancers
Brenner et al., 2001 [24]	Israel	ICR 1983–1999	M—35 F—32	<50 yrs.—5 50–59 yrs.—10 60–69 yrs.—11 70–79 yrs.—29 >80 yrs.—12	3.6 years	5 (total)	2.8 (1.38–4.22)	12 (NA)

Howard et al., 2006 [25]	USA	SEER 1986–2002	M—756 F—560	<60 yrs.—172 60–69 yrs.—246 >70 yrs.—888	3.5 years	83 (after 1 yr.) 122 (total)	1.07 (0.85–1.33)	71 (after 1 yr.) 101 (total)

Koljonen et al., 2010 [26]	Finland	FCR 1979–2006	M—53 F—119	<29 yrs.—1 30–44 yrs.—3 45–59 yrs.—14 60–74 yrs.—50 >75 yrs.—104	4.1 years	1 (with MCC) 33 (after ≥1 m) 34 (total)	1.69 (0.85–3.03)	30 (NA)

Bzhalava et al., 2011 [27]	Denmark Norway Sweden	NCR 1980–2007 1990–2007 1990–2007	M—314 F—442	<29 yrs.—2 30–44 yrs.—8 45–59 yrs.—35 60–74 yrs.—181 >75 yrs.—530	3.5 years	79 (after 6 m) 65 (after 1 yr.) 142 (total)	1.13 (0.89–1.44)	75 (after 6 m) 63 (after 1 yr.)

Youlden et al., 2014 [28]	Australia	QCR 1982–2010	M—512 F—275	75 (29–104)	2.2 years (median)	135 (after 2 m) 105 (after 1 yr.) 240 (total)	1.87 (1.53–2.26)	85 (after 2 m) 63 (after 1 yr.) 148 (total)

NCR: National Cancer Registries; SEER: Surveillance, Epidemiology, and End Results; FCR: Finnish Cancer Registry; NIS: National Institute of Statistics; ICR: Israel Cancer Registry; QCR: Queensland Cancer Registry. SIR: standardized incidence ratio; CI: confidence interval.

**Table 2:** Risk estimates individual second malignancies occurring after one year of MCC diagnosis.

Study, year	Colon cancer	Breast cancer	Lung cancer	NHL	CLL	Malignant melanoma
Howard et al., 2006 [25]	1.48 (0.76–2.58)	0.83 (0.30–1.81)	1.14 (0.61–1.95)	1.65 (0.53–3.85)	1.18 (0.02–6.56)	1.39 (0.28–4.07)
Koljonen et al., 2010 [26]	3.33 (0.40–12.0)	1.43 (0.04–7.95)	—	—	19.7 (0.50–109)	—
Bzhalava et al., 2011 [27]	0.63 (0.26–1.53)	1.02 (0.42–2.45)	1.21 (0.5–2.91)	0.7 (0.1–4.97)	—	3.58 (1.49–8.6)
Youlden et al., 2014 [28]	1.83 (1.21–2.67)	1.76 (0.64–3.82)	1.71 (0.82–3.14)	2.19 (0.60–5.61)	0.00 (0.00–3.83)	3.31 (1.89–5.37)

NHL: non-Hodgkin's lymphoma; CLL: chronic lymphocytic lymphoma.
